# Stealing the spotlight: CUL4-DDB1 ubiquitin ligase docks WD40-repeat proteins to destroy

**DOI:** 10.1186/1747-1028-2-5

**Published:** 2007-02-06

**Authors:** Leigh Ann Higa, Hui Zhang

**Affiliations:** 1 Yale University School of Medicine, Department of Genetics, 333 Cedar Street, New Haven, Connecticut 06520, USA

## Abstract

Recent investigation of Cullin 4 (CUL4) has ushered this class of multiprotein ubiquitin E3 ligases to center stage as critical regulators of diverse processes including cell cycle regulation, developmental patterning, DNA replication, DNA damage and repair, and epigenetic control of gene expression. CUL4 associates with DNA Damage Binding protein 1 (DDB1) to assemble an ubiquitin E3 ligase that targets protein substrates for ubiquitin-dependent proteolysis. CUL4 ligase activity is also regulated by the covalent attachment of the ubiquitin-like protein NEDD8 to CUL4, or neddylation, and the COP9 signalosome complex (CSN) that removes this important modification. Recently, multiple WD40-repeat proteins (WDR) were found to interact with DDB1 and serve as the substrate-recognition subunits of the CUL4-DDB1 ubiquitin ligase. As more than 150–300 WDR proteins exist in the human genome, these findings impact a wide array of biological processes through CUL4 ligase-mediated proteolysis. Here, we review the recent progress in understanding the mechanism of CUL4 ubiquitin E3 ligase and discuss the architecture of CUL4-assembled E3 ubiquitin ligase complexes by comparison to CUL1-based E3s (SCF). Then, we will review several examples to highlight the critical roles of CUL4 ubiquitin ligase in genome stability, cell cycle regulation, and histone lysine methylation. Together, these studies provide insights into the mechanism of this novel ubiquitin ligase in the regulation of important biological processes.

## Background

Ubiquitin-mediated proteolysis has been established as a key regulatory mechanism governing almost every biological process in the eukaryotic cell. Ubiquitination involves the covalent attachment of a polyubiquitin chain to a lysine residue in a substrate protein, and proceeds via three distinct enzymatic activities. Following ATP-dependent ubiquitin activation by ubiquitin activating enzyme (E1) and ubiquitin transfer to an ubiquitin conjugating enzyme (E2), ubiquitin is attached to substrate with the aid of an ubiquitin ligase, or E3. Since E3s interact with substrate directly, the dynamic regulation of E3 activity and substrate specificity is an area of extensive exploration.

CUL4 is a member of the cullin family of proteins, which share substantial homology to CUL1 originally identified in *Caernorhabditis elegans *[1]. Cullins are evolutionarily conserved from yeast to mammals; sequence homology spans the entire protein but is highest at the carboxy (C) terminus, characterized by the ~200 amino acid (AA) cullin homology domain [2]. Humans encode multiple cullins (CUL1, CUL2, CUL3, CUL4A, CUL4B, CUL5, and CUL7) and cullin-like proteins (PARC and APC2) [2]. CUL4 is absent in *Saccharomyces cerevisiae*, but present in *Schizosaccharomyces pombe *(Pcu4), *Xenopus laevis*, *Caenorhabditis elegans*, *Drosophila melanogaster*, and *Arabidopsis thaliana*. Ancestral duplication yielded the mammalian-specific CUL4A and CUL4B, which are over 80% homologous [1]. While CUL4A and CUL4B expression profiles are similar in human tissues [3], CUL4B possesses an unique amino (N) terminal extension of largely unknown function. As shown for CUL1 and CUL3 [2], targeted disruption of the mouse CUL4A gene results in embryonic lethality [4]. The decreased recovery of viable heterozygotes also indicates CUL4A is haploinsufficient, distinguishing it from the rest of the cullin family [4].

Two studies initially established CUL4 ubiquitin E3 ligase activity as a modulator of key biological processes. Zhong et al. [5] found that inactivation of CUL4 in *C. elegans *led to massive rereplication of the genome and the accumulation of giant nuclei containing up to 100C DNA content in certain cells. Immunostaining suggested that replication licensing protein CDT1 was inappropriately stabilized in S phase, and loss of one genomic copy of CDT1 suppressed nuclear polyploidy. This suggested that CUL4 might regulate replication licensing through CDT1 degradation. CDT1 is a subunit of the pre-replication complex and is recruited to replication origins by the origin recognition complex (ORC) and Cdc6 [6]. CDT1 in turn recruits the minichromosome maintenance hexamer MCM2-7 that acts as replicative helicase to license origins. Once MCM is loaded on chromatin, the origin is licensed for DNA synthesis in S phase. CDT1 is also degraded in S-phase to prevent relicensing of fired origins.

Independently, Higa et al. [7] reported that CDT1 is rapidly proteolyzed in response to ultraviolet (UV) and gamma-irradiation (IR). This followed their earlier finding that loss of geminin, an inhibitor of CDT1, led to the CDT1-dependent rereplication and giant polyploid nuclei [8]. Inactivation of CUL4, the RING finger protein ROC1, or CSN subunits, suppressed CDT1 degradation in response to DNA damage in both Drosophila and human cells. Furthermore, CUL4 physically interacts with CDT1 and the isolated CUL4 E3 ligase can polyubiquitinate CDT1 in vitro. These genetic and biochemical studies established that CUL4-ROC1 ubiquitin E3 directly targets CDT1 for degradation in S phase or after UV or IR.

### Comparison of SCF prototype and CUL4

Although CUL4 was implicated in CDT1 degradation and other biological processes (see below), the composition and structure of CUL4 E3 ligase was only recently characterized. However, the homology between cullin E3 ligases suggest that the overall strategy of CUL4 E3 ligase for substrate selection may resemble that of SCF, or CUL1-assembled E3s, which usually serve as an architectural prototype for the rest of cullin family. SCF is named for three of its subunits: SKP1, CUL1/Cdc53, and an F-box protein [2]. The fourth subunit, the small RING finger protein ROC1/RBX1/HRT1, cooperates with all cullins to recruit and activate E2.

CUL1 organizes the substrate receptor and E2 recruitment modules at its amino (N) and carboxy (C) termini, respectively. Structurally, CUL1 forms a stalk-like structure at its N terminus, linked to a C terminal globular domain [9]. The N terminus consists of three cullin repeats, each repeat consisting of a five-helix bundle [9]. CUL1 recognizes substrates through the assembly of a substrate receptor complex, composed of SKP1 and an F-box protein [2]. The N terminal BTB/POZ domain of the invariant adaptor SKP1 interacts with the first cullin repeat of CUL1 (and CUL7) [9,10]. Direct substrate recognition by SCF is performed by the variable component of the substrate receptor, an F-box protein. The F-box is a ~40 AA domain that binds SKP1, and is usually located in the F-box protein adjacent to a protein-protein interaction domain (ie: WD40 repeats or leucine-rich repeats) for substrate binding [11,12]. Thus, F-box proteins are tethered to the SCF core by SKP1 and recruit a spectrum of substrates through additional domains. E2 recruitment by all cullins relies on its tight interaction with the small RING finger protein ROC1 [2]. ROC1 is an essential and highly conserved RING-H2 protein, and harbors the ~70 AA RING finger domain. Through its RING finger, ROC1 is able to recruit and activate E2.

DDB1 was found to interact with CUL4A [13] and was subsequently shown to be the adaptor for CUL4 E3 ligase [14]. As an adaptor protein of CUL4 ligase [14-18], loss of DDB1 compromises the proteolysis of a spectrum of CUL4 substrates in vivo, including CDT1 in response to UV [19-21], and CUL4 ubiquitination activity in vitro [14,19]. DDB1 is evolutionarily conserved from yeast to mammals, and is essential in mammals and Drosophila [20,22] but not yeast [23]. In fission yeast, Rik1 is a putative DDB1 ortholog on the basis of 21% sequence identity and copurification with Pcu4 [24].

Unlike every other cullin adaptor protein, DDB1 does not contain the BTB/POZ fold but is formed by a cluster of three beta propellers (BPA, BPB, BPC), spanning 100 angstroms [25]. BPA and BPC resemble the two halves of an open clamshell, which form a binding pocket. While mutational analysis of CUL1 demonstrated that the rigid orientation between its N and C termini is essential for activity [9], BPB in DDB1 is joined to the BPA-BPC binding pocket by a flexible hinge. Indeed, three different conformations of the BPA-BPC clamshell in relation to the cullin-binding BPB have been observed [15,25], and it will be important to identify what factor(s) can induce such changes, and the impact on ubiquitination activity. Secondly, the CUL4A-DDB1 interface involves two distinct sites, compared to the single CUL1-SKP1 interface [15]. The first interface involves the top surface of DDB1 BPB, notable for seven residues that are identical in all DDB1 orthologs, and the second and fifth helices of the first cullin repeat of CUL4A [15,25]. Despite the significant structural difference between SKP1 and DDB1, this interface is identical to that between SKP1 and CUL1 [15]. The second CUL4A-DDB1 interface occurs between a unique N terminal domain of CUL4A and a peripheral region of the DDB1 BPB [15].

### WD40 repeats define the class of CUL4-DDB1 substrate targeting subunits

Two WD40-repeat proteins, DDB2 and the Cockayne syndrome protein, CSA, have been shown to associate with DDB1, CUL4A, and CSN in mammalian cells [26]. Higa et al. [17,27] and Jin et al. [18] recently found that human WDR protein CDT2 (also known as L2DTL [27] and DTL [28]) associated with CUL4B and DDB1 complexes in vivo and regulated CDT1 proteolysis in response to DNA damage and replication. The *S. pombe *CDT2 was independently identified as CSN2-associated protein that binds to Pcu4 [29]. These observations, together with the observation that human CUL4 [17] and DDB1 [15,16,18] associated with multiple WDR proteins, and the demonstration that each WDR protein performs an unique CUL4-DDB1-dependent function [17,18] led to the establishment of WD40-repeat proteins as substrate-targeting subunits of CUL4-DDB1 ubiquitin E3 ligases. The WD40 repeat spans 40–60 AA, and is notable for a tryptophan-aspartic acid (WD) dipeptide at the C terminus [30]. The average WD40 repeat protein has seven WDRs, with the range being four to sixteen. The basic structural unit of each WD40 repeat is four antiparallel beta strands, which form beta propellers from the last beta strand of the first WD repeat and the first three beta strands of the second repeat. The striking conservation of the WD repeat throughout evolution suggests that the expansive surface area provided by the beta propeller structure is useful to interact, organize, and stabilize multiprotein complexes.

**Figure 1:**
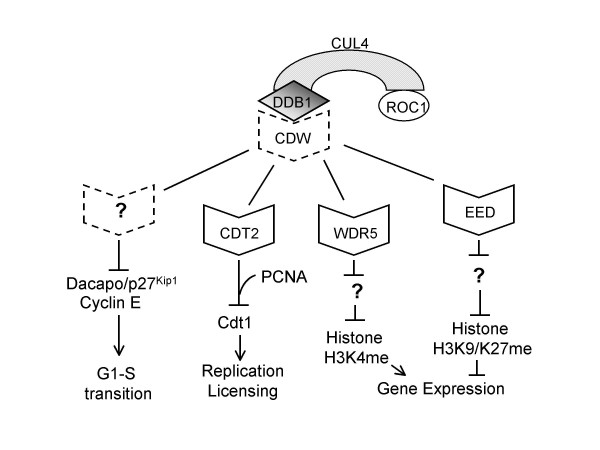
The CUL4-DDB1-CDW ubiquitin E3 ligase. The structure and some biological functions of CUL4-DDB1-CDW ubiquitin E3 ligase are shown. CDW: CUL4-DDB1-associated WD40 repeat proteins.

The majority of WD40 proteins shown to bind DDB1 bear a tandem repeat containing DXXXR/KXWDXR/K (D: Aspartic acid; R/K: Arginine or Lysine; W: Tryptophan; part of this region is also known as the WDXR, DXR, or DWD boxes) [15-18]. The significance of arginine 273 in the WDXR submotif in DDB2 is underscored by its mutation to histidine (R273H) in a xeroderma pigmentosum (XP) group E patient and its failure to bind to DDB1 [13]. Modeling of different WD40 repeat proteins revealed that this arginine (or lysine) is exposed on the bottom face of the barrel-shaped beta propeller structure, and mutation of this residue in either the first or second WDXR submotif severely reduced DDB1 binding in vitro [15-18]. However, a subset of WDR proteins able to bind DDB1 in vivo lack this motif, suggesting that additional motifs can also define DDB1 interaction [15-18]. With hundreds (>150–300) of WDR proteins encoded in the human genome, CUL4-DDB1-ROC1 might regulate a spectacular scope of cellular processes [16,17]. To date, 49 WDR proteins have been shown to interact with CUL-DDB1 in vivo, most by transient overexpression in mammalian cell lines [15-18]. We have proposed to name this class of CUL4 substrate targeting subunits as CUL4 and DDB1-associated WD40-repeat proteins, or CDWs [17] (Figure 1). We would like to propose to name the DXXXR/KXWDXR/K and the related WDR-DDB1 interacting motifs as CDW box. In the following section, we will highlight several cellular processes subject to control by CUL4-DDB1-CDW ubiquitin E3 ligases and discuss the functions of this unique class of ubiquitin E3 ligases.

### Genomic integrity: DNA damage, DNA repair, and DNA replication

Genomic integrity is maintained by mechanisms that integrate the detection and repair of lesions with faithful duplication of the genome. Failure to detect or correct DNA damage, as well as loss of checkpoint mechanisms that prevent entry into S phase, can result in the propagation of mutations. Conversely, loss of the mechanisms that restrict DNA replication to occur once and only once per cell cycle can alter chromosome ploidy and produce genomic instability.

The replication licensing factor CDT1 illustrates how DNA damage signaling intersects with cell cycle control of DNA replication. CDT1 is a subunit of the pre-replication complex [6]. CDT1 and its associated replication licensing activity are primarily regulated by two redundant mechanisms: the specific inhibitor geminin [8], and ubiquitin-mediated proteolysis [31]. Geminin is only found in eukaryotes, and its tight association with chromatin-bound CDT1 in S phase until anaphase occludes the MCM binding site [32]. CDT1 is degraded after DNA damage regardless of cell cycle phase to prevent replication in the presence of damaged DNA [7], and in unperturbed S, G2 and M phases to prevent rereplication [33].

Following UV or IR, CDT1 is rapidly proteolyzed via an ATM/ATR- and CHK1/CHK2-independent pathway [7]. CDT1 proteolysis is evolutionarily conserved between yeast, worms, frogs, flies, and mammals, and is performed by a CUL4-DDB1-CDT2-ROC1 E3 ligase [5,7,14,18,21,34,35]. In mammals, both CUL4A and CUL4B target CDT1 for degradation because both isoforms must be inactivated by small interfering RNA (siRNA) to detect CDT1 stabilization [7,14]. CDT1 proteolysis in G1 cells correlates with a delay in S phase entry, suggesting that CDT1 proteolysis constitutes a checkpoint for DNA replication in G1 [7].

Mammals possess two redundant proteolytic pathways for CDT1: CUL4-DDB1-CDT2 and SCF^SKP2^. Upon S phase entry, CDT1 is phosphorylated on threonine 29 by cyclin E- and cyclin A/CDK2 and directed to SCF^SKP2 ^for proteolysis [36,37]. Alternatively, a PCNA interaction motif (see below) directs CDT1 to a CUL4-DDB1-CDT2 E3 ligase in S/G2 phases and after DNA damage [34,38-40]. However, CUL4-DDB1-mediated CDT1 regulation appears to be conserved among lower metazoans [41].

Although CDT1 is proteolyzed after DNA damage or in S phase, soluble CDT1 can be detected in association with CUL4-DDB1 E3 complex but this pool of CDT1 is not ubiquitinated [34]. CDT1 destruction by CUL4-DDB1-CDT2 is activated by its selective recruitment to chromatin by proliferating cell nuclear antigen (PCNA) [34]. Deletion mutant analysis of *X. laevis *and human CDT1 led to the identification of a PCNA-interaction-protein motif (PIP box) at the extreme N terminus [18,34,42]. PCNA can also be detected in CDT2 (L2DTL) immunoprecipitates [27]. Following origin activation and the recruitment of the enzymatic machinery for DNA synthesis, PCNA is loaded on chromatin and acts as a processivity factor for DNA polymerase. The chromatin association of CDT1 and CUL4-DDB1-CDT2 parallels the kinetics of PCNA loading in S phase in Xenopus extract [18,34]. Consistent with the architecture of the substrate receptor, DDB1 association with chromatin is severely compromised when CDT2 or CDT1 are immunodepleted, or the CDT1 PIP box is disrupted [18,34]. CDT1 bearing a mutated PIP box or loss of CDT2 also induces rereplication in S or G2 phase [34]. Thus, PCNA orchestrates successful origin firing with inhibition of subsequent relicensing by mediating the ubiquitination of CDT1 [34], although how CUL4 E3 is specifically activated on chromatin remains a key point of future investigation. One possibility is that soluble CUL4-DDB1-CUL2 complexes are held in check by CSN, particularly in light of CUL4's targeting to chromatin being correlated with dissociation of CSN after UV irradiation (see below) [26].

Work in *S. pombe *has indicated that CUL4-DDB1-CDT2 is also responsible for CDT1 degradation and is a critical integrator of a DNA damage response pathway distinct from NER and mismatch repair [29,43,44]. This DNA damage response consists of 2 branches, one for moderate genotoxic stress and the other for greater levels of DNA damage [43]. The key effector of the moderate stress branch is Spd1, an inhibitor of ribonucleotide reductase (RNR) assembly. Spd1 prevents RNR activation by sequestering the small subunit of RNR in the nucleus, away the large subunit in the cytoplasm [44]. However, S phase entry and DNA damage drive RNR assembly and activity in the cytoplasm by targeting Spd1 for proteolysis in a Pcu4-DDB1-CDT2 and CSN-dependent pathway [29,44].

Through its regulation of RNR, Spd1 was identified upstream of translesion synthesis in the moderate stress pathway [43]. In contrast, the extreme stress branch is mediated by CUL4 and DDB1 independent of CSN but is equally important for genomic stability because the mutator phenotype of ddb1 null yeast can only be partially suppressed by Spd1 deletion [43].

### Nucleotide excision repair (NER)

The assembly of CUL4-DDB1-ROC1 with two different WD40 repeat proteins, DDB2 and CSA, determines NER pathway activation after UV irradiation [26]. Upon exposure to UV, CUL4A exploits both the substrate targeting and substrate properties of DDB2 to trigger global genome repair [26,45]. In the absence of UV, UV-DDB exists in a CUL4A-ROC1 complex that is inhibited by CSN [26,45]. Immediately after UV, the UV-DDB E3 (CUL4A-DDB1-DDB2-ROC1) is activated by dissociation from CSN and neddylation [26], and recruited to chromatin by DDB2. On chromatin, DDB2 recruits the repair factor XPC, and both DDB2 and XPC are ubiquitinated [45]. Ubiquitinated DDB2 is subsequently degraded, but ubiquitinated XPC is specifically retained on chromatin and deubiquitinated over time [45]. XPC polyubiquitination is required for NER, as addition of methylated ubiquitin which cannot form ubiquitin-ubiquitin conjugates, inhibits NER in cellular extract [45]. In vivo, mutation of R273 in one of the WDXR sequences in DDB2 has been reported in an XP patient, and fails to bind DDB1 [18].

The later activation of transcription-coupled repair after UV depends on the assembly of CSA with CUL4-DDB1-ROC1. CSA (Cockayne Syndrome A) and CSB (Cockayne Syndrome B), define the two complementation groups of Cockayne syndrome [46]. CSA bears seven WD40 repeats; a mutation mapping to one of the WDXR-containing WD40 repeats has been reported and abolishes DDB1 binding in vitro [18]. In vitro, a CUL4-DDB1-CSA-ROC1 complex exhibits ubiquitination activity against recombinant CSB [47]. In vivo, CSA-null cells fail to degrade CSB and the restoration of transcription after transcription-coupled repair does not occur. Interestingly, both CSA and CDT2 interact with the DDB1 BPC but through distinct residues, suggesting that DDB1 recruits distinct CDWs through distinct domains [18].

### Cell cycle progression

Progression through the cell cycle depends on the timely and ordered elimination of both positive and negative regulators of cyclin-dependent kinases (CDKs). CDK activation requires its association with a cyclin subunit, and cyclin protein levels are the net result of synthesis and ubiquitin-mediated proteolysis. CDK activation and assembly is also negatively regulated by cyclin-dependent kinase inhibitors (CDKIs) [48].

SCF is best characterized for its control of the G1/S transition, and SCF-mediated destruction of both G1 cyclins and CDKIs is evolutionarily conserved [49]. Two key targets of SCF, cyclin E and the CDKI p27^Kip1^, are also subject to control by CUL4-DDB1 [21]. Inactivation of CUL4 in Drosophila, and CUL4A alone in mammals, produces G1 arrest which can be counteracted by concomitant loss of Dacapo (the single Drosophila CDKI) or p27 by RNA interference (RNAi). Cyclin E also accumulates after Drosophila CUL4 or human CUL4A and CUL4B RNAi. Phosphorylation-dependent, p27 degradation by SCF^Skp2 ^can be recapitulated in mammalian cell extracts supplemented with cyclin E/CDK2 [50]. Cell extracts depleted of CUL4A or DDB1 by siRNA still possess p27 degradation activity, suggesting that SCF^SKP2 ^and CUL4A-DDB1 act independently [21]. In contrast, SKP2 has also been reported to interact with CUL4-DDB1 by coupled transfection and coimmunoprecipitation assays [51]. While this observation suggests that the leucine-rich repeat protein SKP2 instead serves as the substrate targeting subunit for p27, this study also demonstrated that CUL1 and CUL4 act independently because CUL1 ablation by siRNA in mammalian cells did not affect p27 protein stability after adenoviral infection with DDB1 [51]. Furthermore, since several F-box proteins with WD40 repeats (mouse FBXW1/beta-TRCP, FBXW5, and FBXW8), can bind DDB1 in vitro [16], the significance of such CUL4-F-box-protein assemblies should be investigated.

Besides the identification of the CDW(s) that recognize p27 and cyclin E, several open questions about CUL4 structure and regulation remain. First, what determines whether a substrate is recognized by CUL4A, CUL4B, or both? Secondly, what structural features of CUL4 specify its differential regulation by CAND1, CSN, and neddylation? CAND1 was identified as an inhibitor of SCF assembly [52,53], and its interaction with the cullin family is regulated by the cullin neddylation/deneddylation cycle [52-54]. Ablation of Drosophila CAND1 by RNAi greatly affects armadillo (the Drosophila homolog of beta-catenin) protein levels but not cyclin E, while CSN5 RNAi has the opposite effect [21]. Since cyclin E is a target of both SCF^Fbw7/Ago ^and CUL4-DDB1, this suggests that CUL4 deneddylation is particularly critical for proper function in vivo. Third, what degron sequences besides the PIP box, or post-trasnslational modifications, specify CUL4 substrate recruitment? In this regard, a high molecular weight isoform of Drosophila cyclin E accumulates after CUL4 RNAi. Furthermore, Dacapo, but not mammalian p27, contains a PIP box [55]. The Xenopus CDK inhibitor Xic1 is recruited to PCNA by its PIP box, and its ubiquitination on chromatin after replication initiation is independent of CDK2-cyclin E mediated phosphorylation [56]. Finally, how are SCF and CUL4 E3 activity coordinately regulated for their common substrates? Vertebrate p27 and Xic1 (but not Dacapo), cyclin E and CDT1 are all regulated by SCF and CUL4 [7,21,31,57].

CUL4A-DDB1-CDT2-ROC1 also interacts with and targets the tumor suppressor protein p53, which mediates cell cycle arrest or apoptosis in the face of genotoxic stress [58]. In an unstressed cell, p53 protein levels are kept low through proteolysis, and the best characterized pathway for p53 stability includes MDM2. MDM2 (HDM2 in human) is a variant RING finger protein, and has been shown in vitro to catalyze monoubiquitination, but not polyubiquitination, of p53. In vivo, MDM2 is required for p53 proteolysis, leading to the suggestion that p53 ubiquitination by MDM2 requires other cellular factors [59]. CUL4A complex may serve such a role for p53 degradation. While loss of any component of the CUL4A-ROC1-DDB1-CDT2 complex by specific siRNAs led to the stabilization of p53 protein [19], overexpression of CUL4A is sufficient to downregulate p53 [60]. Consistent with these data, in vivo deletion of DDB1 in mice causes p53 accumulation [20]. MDM2's incorporation into the CUL4-DDB1-CDT2-ROC1 E3 complex is essential for p53 turnover by CUL4: isolated CUL4 complexes displayed robust ubiquitination activity against p53 in vitro which is severely diminished in MDM2-/- MEFs, and can be restored by expression of wild type MDM2 [19]. Depletion of DDB1 or CDT2 abolishes CUL4 ubiquitination activity for p53. Furthermore, inactivation of PCNA by siRNA dramatically increases p53 protein levels in the absence of DNA damage, and PCNA interacts tightly with MDM2, as well as p53. MDM2 contains a recognizable PIP box at its extreme C terminus, but the ability of a MDM2 PIP-box deletion mutant to still bind PCNA indicates that additional domains likely exist [19]. Indeed, the selective association of MDM2 and PCNA after UV (but not IR) triggers MDM2 proteolysis, and is accompanied by dissociation of p53 from CUL4A, which raises the question of how recognition of the constitutive PIP box degron is accomplished. CDT1, MDM2 and p53 all contain the PIP box, but CDT1 and MDM2 are degraded after UV, while p53 is stabilized [19]. The presence of MDM2 as a second RING finger protein in the CUL4A-ROC1 E3 complex is also intriguing, and the functional relationship between MDM2 and ROC1 in E2 recruitment and polyubiquitin chain formation is being further investigated. Interestingly, COP1, a WDR and RING finger protein, was also found to regulate p53 protein stability by interacting with p53 independent of MDM2 [61]. Recently, it was found that COP1 interacts with CUL4-DDB1 in vivo [17], suggesting that CUL4-DDB1 may also participate in COP1-mediated processes.

### Histone modification

Chromatin dynamics is determined by two distinct mechanisms: post-translational modification of histone proteins, and ATP-dependent nucleosome remodeling. Both processes act on the nucleosome, the fundamental unit of chromatin that consists of ~146 bp of DNA wrapped around a histone octamer (a tetramer of histone H2A and H2B and a tetramer of histone H3 and H4). The globular domain of histones interacts with DNA, while a flexible tail domain protrudes from the nucleosome and is subject to multiple modifications, including phosphorylation, acetylation, ubiquitination, and methylation [62]. The number and type of histone modifications are translated into the alteration of chromatin structure which underlie the temporal and spatial control of gene expression, heterochromatin formation, DNA replication, and DNA repair [63]. Recent studies indicate that CUL4-based E3 ligases regulate histone lysine methylation associated with both gene activation and gene silencing [17,64].

The WDR proteins WDR5 and RBBP5 are essential components of the MLL (mixed-lineage-leukemia) histone methyltransferase complex specific for histone H3 methylation on lysine 4 (H3K4) [65]. H3K4 can be mono- (me^1^), di- (me^2^), or tri-methylated (me^3^), and tri-methylated H3K4 marks almost all active transcription promoters and is part of epigenetic control. Loss of WDR5 severely abrogates H3K4me^1 ^and me^3^, with less effect on H3K4me^2^. WDR5 inactivation by siRNA in mammalian cells reduced HOX gene expression, and injection of *X. laevis *embryos with WDR5 morpholino resulted in severe developmental abnormalities. Consistent with its identification as a specific H3K4me^2^- and H3K4me^3^-binding protein, WDR5 is postulated to stimulate the conversion of H3K4me^2 ^to H3K4me^3^.

Higa et al. (2006) found that CUL4-DDB1 interacts with WDR5 and RBBP5 and regulates histone H3 methylation at K4 [17]. Like WDR5 and RBBP5, inactivation of CUL4 or DDB1 by siRNAs leads to drastic loss of H3K4me^3 ^and H3K4me^1 ^[17]. In vivo, DDB1 and CUL4A can be detected in H3K4 mono-, di-, and tri-methylated H3K4 mononucleosomes. CUL4-DDB1 also associates with H3K4me^3 ^but not unmodified H3 peptide in vitro. The interactions between WDR5 or RBBP5 and CUL4-DDB1 and the functional involvement of CUL4-DDB1 ubiquitin ligase in H3K4 methylation support the notion that ubiquitin-dependent proteolysis underlies the dynamic regulation of epigenetic marks at H3K4. The ubiquitin ligase activity of CUL4-DDB1 assembled with WDR5 and RBBP5 may thus target a negative regulator of H3K methylation. On the other hand, the demonstration in this study that distinct WDR proteins, WDR5 and CDT2 (L2DTL), perform distinct biological functions through CUL4-DDB1 ligase supports the notion that CDW identity differentiates the biological processes influenced by CUL4-DDB1-ROC1 ubiquitination. Thus maintenance of CDT1 destruction after DNA damage in WDR5-deficient cells and maintenance of H3K4 methylation in CDT2-silenced cells established that WDR proteins are substrate-targeting subunits of CUL4-DDB1 ubiquitin ligases.

Polycomb group (PcG) proteins maintain the silenced state of homeotic genes [62]. Higa et al. (2006) discovered that CUL4-DDB1 binds the polycomb group protein EED (embryonic ectoderm development, mammalian homologue of Drosophila ESC, Extra sex comb), a WD40-repeat subunit of the EZH2 (Enhancer of Zeste homologue 2) methyltransferase complex [17]. The EED-EZH2-SUZ12 methyltransferase directs histone H3 methylation at lysine 9 and lysine 27 (H3K9 and H3K27) for X chromosome inactivation, genomic imprinting, heterochromatin formation, and gene silencing [62]. The specific association of CUL4-DDB1 with tri-methylated H3K9 and H3K27 mononucleosomes was also observed [17]. Inactivation of CUL4 or DDB1 significantly abolished tri-methylation at K9 and K27, suggesting this ligase complex is functionally involved. Thus destruction of H3K9/K27-specific regulators by CUL4-DDB1 ubiquitin E3 ligase might regulate these epigenetic processes [17].

In fission yeast, Pcu4-Rik1 regulates H3K9 methylation through its recruitment of the Clr4 methyltransferase to chromatin and this is independent of DDB1 [64]. A requirement for CUL4 catalytic activity was shown in *S. pombe*, where a Pcu4 non-neddylated mutant could not restore gene silencing to Pcu4 null yeast [64]. Potential targets of CUL4 ubiquitination include antagonists of histone methylation [66] or histones themselves. In yeast, mammals, and likely Drosophila, monoubiquitination of histone H2A/H2B is a prerequisite for histone H3/H4 methylation [67]. Given the tight coupling of H2A and H2B ubiquitination to transcriptional activation, determining if CUL4 might regulate transcription is worthy of further study. Finally, how is CUL4 activity programmed to accomplish monoubiquitination or polyubiquitination? DDB2 has been implicated in H2A ubiquitination [68], and CUL4-DDB1-ROC1 devoid of CSN has been shown to ubiquitinate H3 and H4 in vitro to promote repair [69]. It will be interesting to analyze the contribution of E2 specificity to the processivity of CUL4 ubiquitination, since the association of the Polycomb PRC1 E3 ligase with different UbcH5 E2 isoforms has been shown to influence H2A ubiquitination [70].

## Conclusion

While our knowledge of CUL4 E3 ligases has expanded significantly, many provocative questions remain. One critical question is whether all WDR proteins or a subset of them bind to CUL4-DDB1 and perform a function in protein ubiquitination. CUL4 function in vivo should also be addressed with genetic analysis in knockout animals or cells.

Since CUL4A amplification has been noted in breast and hepatocellular cancer [71,72], it will be interesting to determine if CUL4A/4B gene dosage alters tumor progression in vivo. The flexible nature of the DDB1 adaptor protein, as well as chromatin-templated ubiquitination, suggest that CUL4's ability to recognize, stably bind, and polyubiquitinate substrates is exquisitely regulated. Beyond identifying new targets for CUL4-DDB1-CDW, investigation of these regulatory mechanisms promises to keep CUL4 E3s in the spotlight for the foreseeable future.

## Competing interests

The author(s) declare that they have no competing interests.

## Authors' contributions

L.A.H. and H.Z. both drafted the manuscript and designed the figure. All authors read and approved the final manuscript.

## References

[B1] Kipreos ET, Lander LE, Wing JP, He WW, Hedgecock EM (1996). cul-1 is required for cell cycle exit in C. elegans and identifies a novel gene family. Cell.

[B2] Petroski MD, Deshaies RJ (2005). Function and regulation of cullin-RING ubiquitin ligases. Nat Rev Mol Cell Biol.

[B3] Hori T, Osaka F, Chiba T, Miyamoto C, Okabayashi K, Shimbara N, Kato S, Tanaka K (1999). Covalent modification of all members of human cullin family proteins by NEDD8. Oncogene.

[B4] Li B, Ruiz JC, Chun KT (2002). CUL-4A is critical for early embryonic development. Mol Cell Biol.

[B5] Zhong W, Feng H, Santiago FE, Kipreos ET (2003). CUL-4 ubiquitin ligase maintains genome stability by restraining DNA-replication licensing. Nature.

[B6] Machida YJ, Hamlin JL, Dutta A (2005). Right place, right time, and only once: replication initiation in metazoans. Cell.

[B7] Higa LA, Mihaylov IS, Banks DP, Zheng J, Zhang H (2003). Radiation-mediated proteolysis of CDT1 by CUL4-ROC1 and CSN complexes constitutes a new checkpoint. Nat Cell Biol.

[B8] Mihaylov IS, Kondo T, Jones L, Ryzhikov S, Tanaka J, Zheng J, Higa LA, Minamino N, Cooley L, Zhang H (2002). Control of DNA replication and chromosome ploidy by geminin and cyclin A. Mol Cell Biol.

[B9] Zheng N, Schulman BA, Song L, Miller JJ, Jeffrey PD, Wang P, Chu C, Koepp DM, Elledge SJ, Pagano M (2002). Structure of the Cul1-Rbx1-Skp1-F boxSkp2 SCF ubiquitin ligase complex. Nature.

[B10] Schulman BA, Carrano AC, Jeffrey PD, Bowen Z, Kinnucan ER, Finnin MS, Elledge SJ, Harper JW, Pagano M, Pavletich NP (2000). Insights into SCF ubiquitin ligases from the structure of the Skp1-Skp2 complex. Nature.

[B11] Jin J, Cardozo T, Lovering RC, Elledge SJ, Pagano M, Harper JW (2004). Systematic analysis and nomenclature of mammalian F-box proteins. Genes Dev.

[B12] Bai C, Sen P, Hofmann K, Ma L, Goebl M, Harper JW, Elledge SJ (1996). SKP1 connects cell cycle regulators to the ubiquitin proteolysis machinery through a novel motif, the F-box. Cell.

[B13] Shiyanov P, Nag A, Raychaudhuri P (1999). Cullin 4A associates with the UV-damaged DNA-binding protein DDB. J Biol Chem.

[B14] Hu J, McCall CM, Ohta T, Xiong Y (2004). Targeted ubiquitination of CDT1 by the DDB1-CUL4A-ROC1 ligase in response to DNA damage. Nat Cell Biol.

[B15] Angers S, Li T, Yi X, MacCoss MJ, Moon RT, Zheng N (2006). Molecular architecture and assembly of the DDB1-CUL4A ubiquitin ligase machinery. Nature.

[B16] He YJ, McCall CM, Hu J, Zeng Y, Xiong Y (2006). DDB1 functions as a linker to recruit receptor WD40 proteins to CUL4-ROC1 ubiquitin ligases. Genes Dev.

[B17] Higa LA, Wu M, Ye T, Kobayashi R, Sun H, Zhang H (2006). CUL4-DDB1 ubiquitin ligase interacts with multiple WD40-repeat proteins and regulates histone methylation. Nat Cell Biol.

[B18] Jin J, Arias EE, Chen J, Harper JW, Walter JC (2006). A family of diverse Cul4-Ddb1-interacting proteins includes Cdt2, which is required for S phase destruction of the replication factor Cdt1. Mol Cell.

[B19] Banks D, Wu M, Higa LA, Gavrilova N, Quan J, Ye T, Kobayashi R, Sun H, Zhang H (2006). L2DTL/CDT2 and PCNA interact with p53 and regulate p53 polyubiquitination and protein stability through MDM2 and CUL4A/DDB1 complexes. Cell Cycle.

[B20] Cang Y, Zhang J, Nicholas SA, Bastien J, Li B, Zhou P, Goff SP (2006). Deletion of DDB1 in mouse brain and lens leads to p53-dependent elimination of proliferating cells. Cell.

[B21] Higa LA, Yang X, Zheng J, Banks D, Wu M, Ghosh P, Sun H, Zhang H (2006). Involvement of CUL4 ubiquitin E3 ligases in regulating CDK inhibitors Dacapo/p27Kip1 and cyclin E degradation. Cell Cycle.

[B22] Takata K, Yoshida H, Yamaguchi M, Sakaguchi K (2004). Drosophila damaged DNA-binding protein 1 is an essential factor for development. Genetics.

[B23] Zolezzi F, Fuss J, Uzawa S, Linn S (2002). Characterization of a Schizosaccharomyces pombe strain deleted for a sequence homologue of the human damaged DNA binding 1 (DDB1) gene. J Biol Chem.

[B24] Hong EJ, Villen J, Gerace EL, Gygi SP, Moazed D (2005). A Cullin E3 Ubiquitin Ligase Complex Associates with Rik1 and the Clr4 Histone H3-K9 Methyltransferase and Is Required for RNAi-Mediated Heterochromatin Formation. RNA Biol.

[B25] Li T, Chen X, Garbutt KC, Zhou P, Zheng N (2006). Structure of DDB1 in complex with a paramyxovirus V protein: viral hijack of a propeller cluster in ubiquitin ligase. Cell.

[B26] Groisman R, Polanowska J, Kuraoka I, Sawada J, Saijo M, Drapkin R, Kisselev AF, Tanaka K, Nakatani Y (2003). The ubiquitin ligase activity in the DDB2 and CSA complexes is differentially regulated by the COP9 signalosome in response to DNA damage. Cell.

[B27] Higa LA, Banks D, Wu M, Kobayashi R, Sun H, Zhang H (2006). L2DTL/CDT2 interacts with the CUL4/DDB1 complex and PCNA and regulates CDT1 proteolysis in response to DNA damage. Cell Cycle.

[B28] Sansam CL, Shepard JL, Lai K, Ianari A, Danielian PS, Amsterdam A, Hopkins N, Lees JA (2006). DTL/CDT2 is essential for both CDT1 regulation and the early G2/M checkpoint. Genes Dev.

[B29] Liu C, Poitelea M, Watson A, Yoshida SH, Shimoda C, Holmberg C, Nielsen O, Carr AM (2005). Transactivation of Schizosaccharomyces pombe cdt2+ stimulates a Pcu4-Ddb1-CSN ubiquitin ligase. Embo J.

[B30] Li D, Roberts R (2001). WD-repeat proteins: structure characteristics, biological function, and their involvement in human diseases. Cell Mol Life Sci.

[B31] Fujita M (2006). Cdt1 revisited: complex and tight regulation during the cell cycle and consequences of deregulation in mammalian cells. Cell Div.

[B32] Lee C, Hong B, Choi JM, Kim Y, Watanabe S, Ishimi Y, Enomoto T, Tada S, Kim Y, Cho Y (2004). Structural basis for inhibition of the replication licensing factor Cdt1 by geminin. Nature.

[B33] Nishitani H, Sugimoto N, Roukos V, Nakanishi Y, Saijo M, Obuse C, Tsurimoto T, Nakayama KI, Nakayama K, Fujita M (2006). Two E3 ubiquitin ligases, SCF-Skp2 and DDB1-Cul4, target human Cdt1 for proteolysis. Embo J.

[B34] Arias EE, Walter JC (2006). PCNA functions as a molecular platform to trigger Cdt1 destruction and prevent re-replication. Nat Cell Biol.

[B35] Ralph E, Boye E, Kearsey SE (2006). DNA damage induces Cdt1 proteolysis in fission yeast through a pathway dependent on Cdt2 and Ddb1. EMBO Rep.

[B36] Nishitani H, Taraviras S, Lygerou Z, Nishimoto T (2001). The human licensing factor for DNA replication Cdt1 accumulates in G1 and is destabilized after initiation of S-phase. J Biol Chem.

[B37] Sugimoto N, Tatsumi Y, Tsurumi T, Matsukage A, Kiyono T, Nishitani H, Fujita M (2004). Cdt1 phosphorylation by cyclin A-dependent kinases negatively regulates its function without affecting geminin binding. J Biol Chem.

[B38] Hu J, Xiong Y (2006). An evolutionarily conserved function of proliferating cell nuclear antigen for cdt1 degradation by the cul4-ddb1 ubiquitin ligase in response to DNA damage. J Biol Chem.

[B39] Nishitani H, Sugimoto N, Roukos V, Nakanishi Y, Saijo M, Obuse C, Tsurimoto T, Nakayama KI, Nakayama K, Fujita M (2006). Two E3 ubiquitin ligases, SCF-Skp2 and DDB1-Cul4, target human Cdt1 for proteolysis. Embo J.

[B40] Senga T, Sivaprasad U, Zhu W, Park JH, Arias EE, Walter JC, Dutta A (2006). PCNA is a co-factor for Cdt1 degradation by CUL4/DDB1 mediated N-terminal ubiquitination. J Biol Chem.

[B41] Kim Y, Kipreos ET (2006). The C. elegans replication licensing factor CDT-1 is targeted for degradation by the CUL-4/DDB-1 complex. Mol Cell Biol.

[B42] Senga T, Sivaprasad U, Zhu W, Park JH, Arias EE, Walter JC, Dutta A (2006). PCNA is a cofactor for Cdt1 degradation by CUL4/DDB1-mediated N-terminal ubiquitination. J Biol Chem.

[B43] Holmberg C, Fleck O, Hansen HA, Liu C, Slaaby R, Carr AM, Nielsen O (2005). Ddb1 controls genome stability and meiosis in fission yeast. Genes Dev.

[B44] Liu C, Powell KA, Mundt K, Wu L, Carr AM, Caspari T (2003). Cop9/signalosome subunits and Pcu4 regulate ribonucleotide reductase by both checkpoint-dependent and -independent mechanisms. Genes Dev.

[B45] Sugasawa K, Okuda Y, Saijo M, Nishi R, Matsuda N, Chu G, Mori T, Iwai S, Tanaka K, Hanaoka F (2005). UV-induced ubiquitylation of XPC protein mediated by UV-DDB-ubiquitin ligase complex. Cell.

[B46] Laine JP, Egly JM (2006). When transcription and repair meet: a complex system. Trends Genet.

[B47] Groisman R, Kuraoka I, Chevallier O, Gaye N, Magnaldo T, Tanaka K, Kisselev AF, Harel-Bellan A, Nakatani Y (2006). CSA-dependent degradation of CSB by the ubiquitin-proteasome pathway establishes a link between complementation factors of the Cockayne syndrome. Genes Dev.

[B48] Sherr CJ, Roberts JM (1999). CDK inhibitors: positive and negative regulators of G1-phase progression. Genes Dev.

[B49] Deshaies RJ (1999). SCF and Cullin/Ring H2-based ubiquitin ligases. Annu Rev Cell Dev Biol.

[B50] Tsvetkov LM, Yeh KH, Lee SJ, Sun H, Zhang H (1999). p27(Kip1) ubiquitination and degradation is regulated by the SCF(Skp2) complex through phosphorylated Thr187 in p27. Curr Biol.

[B51] Bondar T, Kalinina A, Khair L, Kopanja D, Nag A, Bagchi S, Raychaudhuri P (2006). Cul4A and DDB1 associate with Skp2 to target p27Kip1 for proteolysis involving the COP9 signalosome. Mol Cell Biol.

[B52] Liu J, Furukawa M, Matsumoto T, Xiong Y (2002). NEDD8 modification of CUL1 dissociates p120(CAND1), an inhibitor of CUL1-SKP1 binding and SCF ligases. Mol Cell.

[B53] Zheng J, Yang X, Harrell JM, Ryzhikov S, Shim EH, Lykke-Andersen K, Wei N, Sun H, Kobayashi R, Zhang H (2002). CAND1 binds to unneddylated CUL1 and regulates the formation of SCF ubiquitin E3 ligase complex. Mol Cell.

[B54] Cope GA, Deshaies RJ (2003). COP9 signalosome: a multifunctional regulator of SCF and other cullin-based ubiquitin ligases. Cell.

[B55] Warbrick E, Heatherington W, Lane DP, Glover DM (1998). PCNA binding proteins in Drosophila melanogaster : the analysis of a conserved PCNA binding domain. Nucleic Acids Res.

[B56] Chuang LC, Yew PR (2005). Proliferating cell nuclear antigen recruits cyclin-dependent kinase inhibitor Xic1 to DNA and couples its proteolysis to DNA polymerase switching. J Biol Chem.

[B57] Lin HR, Chuang LC, Boix-Perales H, Philpott A, Yew PR (2006). Ubiquitination of cyclin-dependent kinase inhibitor, Xic1, is mediated by the Xenopus F-box protein xSkp2. Cell Cycle.

[B58] Brooks CL, Gu W (2006). p53 ubiquitination: Mdm2 and beyond. Mol Cell.

[B59] Grossman SR, Deato ME, Brignone C, Chan HM, Kung AL, Tagami H, Nakatani Y, Livingston DM (2003). Polyubiquitination of p53 by a ubiquitin ligase activity of p300. Science.

[B60] Nag A, Bagchi S, Raychaudhuri P (2004). Cul4A physically associates with MDM2 and participates in the proteolysis of p53. Cancer Res.

[B61] Dornan D, Wertz I, Shimizu H, Arnott D, Frantz GD, Dowd P, O'Rourke K, Koeppen H, Dixit VM (2004). The ubiquitin ligase COP1 is a critical negative regulator of p53. Nature.

[B62] Martin C, Zhang Y (2005). The diverse functions of histone lysine methylation. Nat Rev Mol Cell Biol.

[B63] Jenuwein T, Allis CD (2001). Translating the histone code. Science.

[B64] Jia S, Kobayashi R, Grewal SI (2005). Ubiquitin ligase component Cul4 associates with Clr4 histone methyltransferase to assemble heterochromatin. Nat Cell Biol.

[B65] Wysocka J, Swigut T, Milne TA, Dou Y, Zhang X, Burlingame AL, Roeder RG, Brivanlou AH, Allis CD (2005). WDR5 associates with histone H3 methylated at K4 and is essential for H3 K4 methylation and vertebrate development. Cell.

[B66] Shi Y, Whetstine JR (2007). Dynamic regulation of histone lysine methylation by demethylases. Mol Cell.

[B67] Osley MA, Fleming AB, Kao CF (2006). Histone ubiquitylation and the regulation of transcription. Results Probl Cell Differ.

[B68] Kapetanaki MG, Guerrero-Santoro J, Bisi DC, Hsieh CL, Rapic-Otrin V, Levine AS (2006). The DDB1-CUL4ADDB2 ubiquitin ligase is deficient in xeroderma pigmentosum group E and targets histone H2A at UV-damaged DNA sites. Proc Natl Acad Sci USA.

[B69] Wang H, Zhai L, Xu J, Joo HY, Jackson S, Erdjument-Bromage H, Tempst P, Xiong Y, Zhang Y (2006). Histone H3 and H4 ubiquitylation by the CUL4-DDB-ROC1 ubiquitin ligase facilitates cellular response to DNA damage. Mol Cell.

[B70] Buchwald G, van der Stoop P, Weichenrieder O, Perrakis A, van Lohuizen M, Sixma TK (2006). Structure and E3-ligase activity of the Ring-Ring complex of polycomb proteins Bmi1 and Ring1b. Embo J.

[B71] Chen LC, Manjeshwar S, Lu Y, Moore D, Ljung BM, Kuo WL, Dairkee SH, Wernick M, Collins C, Smith HS (1998). The human homologue for the Caenorhabditis elegans cul-4 gene is amplified and overexpressed in primary breast cancers. Cancer Res.

[B72] Yasui K, Arii S, Zhao C, Imoto I, Ueda M, Nagai H, Emi M, Inazawa J (2002). TFDP1, CUL4A, and CDC16 identified as targets for amplification at 13q34 in hepatocellular carcinomas. Hepatology.

